# A case of female adrenoleukodystrophy carrier with insidious neurogenic bladder

**DOI:** 10.1002/jgf2.314

**Published:** 2020-03-18

**Authors:** Koji Obara, Erika Abe, Nobuyuki Shimozawa, Itaru Toyoshima

**Affiliations:** ^1^ Department of Neurology National Hospital Organization Akita National Hospital Yurihonjo Japan; ^2^ Division of Genomics Research Life Science Research Center Gifu University Gifu Japan

**Keywords:** adrenoleukodystrophy, adrenomyeloneuropathy, clean intermittent catheterization, female carrier, overactive bladder

## Abstract

A 65‐year‐old woman with mutation of the ABCD1 gene for adrenoleukodystrophy (ALD) was admitted to our hospital with a urinary tract infection. Abdominal computed tomography showed dilation of the urinary tract. Although she had noticed pollakisuria since her forties, she had not been followed up by any medical institutions until we diagnosed her as a female carrier with ALD. ALD is an X‐linked pattern of inheritance that typically affects males, but many female carriers actually present slowly progressive myelopathy and neuropathy. Therefore, it is important to identify female carriers with ALD and treat them at the earliest stage possible.

## Graphical abstract

Many female adrenoleukodystrophy carriers manifest signs and symptoms of myelopathy and polyneuropathy. Therefore, it is important to identify female carriers with ALD and treat them at the earliest stage possible.

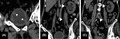

## 1 INTRODUCTION

Adrenoleukodystrophy (ALD) is an X‐linked hereditary disease caused by mutations in the ABCD1 gene that encodes the peroxisomal transporter of very long‐chain fatty acids. The clinical spectrum in males with ALD is varied, including isolated adrenocortical insufficiency, slowly progressive myelopathy, polyneuropathy (adrenomyeloneuropathy: AMN), and devastating cerebral demyelination (cerebral ALD: CALD).^1^ On the other hand, the clinical features of female carriers with ALD are not well known. However, many female carriers with ALD present AMN‐like symptoms.^2^, ^3^ Here, we report the case of a female ALD carrier with dilation of the urinary tract accompanied by urinary dysfunction.

## 2 CASE REPORT

**Figure 1:**
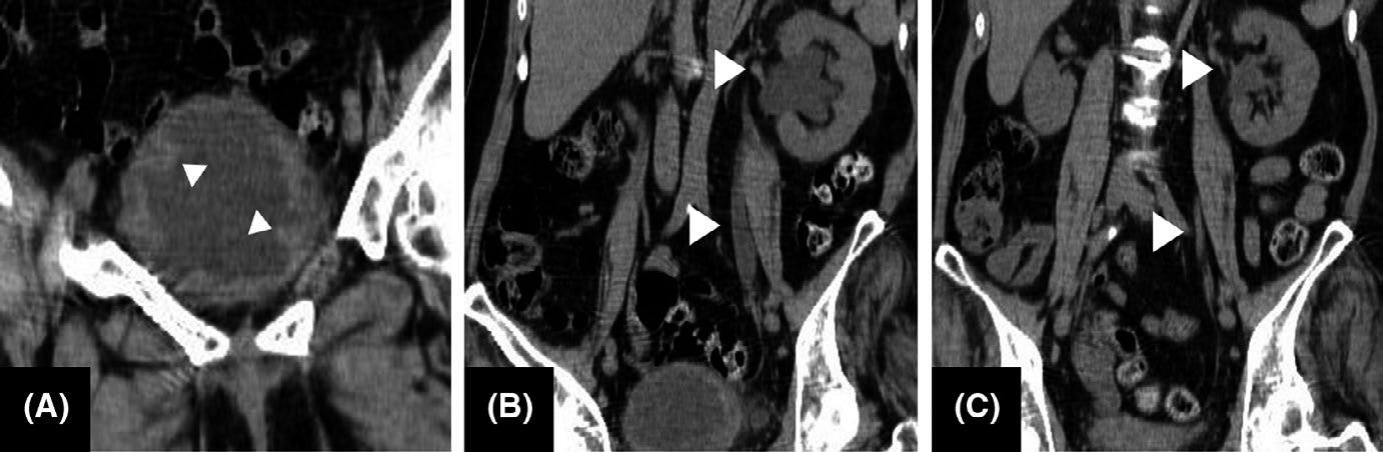
Abdominal CT images taken at admission showed trabeculated bladder (A) and dilation of left renal pelvis and ureter (B). CT image on day 15 showed reduction of dilated urinary tract (C)

The patient is a 65‐year‐old woman who has a brother and a sister. Her brother and a nephew were diagnosed with AMN and CALD, respectively. Her nephew died at 31 years of age. Her sister had a gait disturbance. Since her forties, the patient has also noticed walking difficulty, pollakisuria, and spasticity of both legs. Later, her gait disturbance slowly progressed. She attributed her symptoms to age, despite her family history. At age 60, she visited our hospital for the first time. At that time, she was able to walk with assistance. As the pollakisuria got worse, she experienced incontinence and noticed difficulty in urination, but she had no sensation of residual urine. Referring to her family history, we tested her ABCD1 gene and identified a G298S hemizygous mutation. Hence, we diagnosed her illness as female ALD carrier. Three days before admission, she had a low‐grade fever, a dull ache in the suprapubic region, and malaise. On physical examination, her temperature was 37.4°C. Her suprapubic region was soft, but there was mild tenderness. Knocking pain was not provoked in the costovertebral angle area. On neurologic examination, spasticity with contracture of hip joints, muscle weakness (MMT 2/5), and moderate hypesthesia were observed in bilateral lower limbs. She could stand with assistance. The tendon reflexes of the upper and lower extremities were symmetrically hyperactive except for the Achilles reflex, which was absent, and bilateral Babinski signs were elicited. There were no meningeal signs. Laboratory studies on admission yielded the following values: white blood cell count 9740/mm^3^ with 80% neutrophils, C‐reactive protein (CRP) 3.92 mg/dL, and serum creatinine 0.74 mg/dL. The urine was turbid, the sediment of which was loaded with white cells and bacteria. The urine culture grew *Escherichia coli*. Abdominal computed tomography (CT) images showed trabeculated bladder and dilation of the left renal pelvis and ureter (Figure 1). These findings indicated the presence of urinary stasis due to residual urine and vesicoureteral reflux. She was treated with 500 mg/day of levofloxacin, and her symptoms improved quickly. To decrease her residual urine volume, we introduced clean intermittent catheterization (CIC) three times a day. Even after urination, around 400 mL of urine was sometimes still obtained with a catheter. Follow‐up CT on day 15 of admission showed reduction of the dilated urinary tract (Figure 1). CIC was continued after discharge.

## 3 DISCUSSION

Since ALD has an X‐linked pattern and is classically a disorder affecting only males, it was assumed that female carriers remained asymptomatic.^1^, ^2^, ^3^ However, it is now recognized that many female carriers also manifest symptoms, which include spastic paraparesis, sensory disturbance, and bladder and bowel dysfunction, like males with AMN.^1^, ^2^, ^3^ Symptoms of bladder dysfunction are urinary urgency, nocturia, and incontinence, which are signs of so‐called overactive bladder (OBA),^4^ and are often reported as the initial symptoms recognized by the patient.^3^ A urodynamic study (not done for our patient) demonstrates the presence of detrusor overactivity and detrusor dyssynergia, indicating spinal cord dysfunction.^4^, ^5^ These symptoms in female carriers usually appear in middle age and slowly progress with age.^1^, ^3^ Because knowledge of female ALD is not common among physicians, these symptoms often remain unidentified.^2^ In fact, about 20 years passed from the emergence of the initial symptoms to the first consultation in our patient. In the meantime, voiding difficulty and a backward urine flow to the upper urinary tract might occur slowly. Subsequently, dilation of the urinary tract and infection due to urinary stasis must have developed. It seems that one of the causes of delayed intervention in our patient was a lack of feeling of residual urine due to impaired bladder sensation. CIC is effective even in an advanced stage, as in our patient. Therefore, if we encounter an ALD patient, we should identify possible female carries in his family. Although there is no effective disease‐modifying therapy available yet for female carriers with ALD,^1^ if female carriers are symptomatic, we should intervene as soon as possible. For example, a female carrier could be referred to an urologist for evaluation of insidious neurogenic bladder and initiation of CIC to prevent urinary tract infection and deformity.^1^, ^5^

## ACKNOWLEDGEMENT

None.

## CONFLICT OF INTEREST

The authors declare no conflict of interests for this article.

## INFORMED CONSENT

We have obtained written informed consent from the patient for publication of this case report.
